# Editorial

**DOI:** 10.1002/jha2.75

**Published:** 2020-08-26

In partnership with the British Society of Haematology and Wiley, we are thrilled to publish our inaugural issue of *eJHaem*. The *eJHaem* functions as a cascading, open access journal in close collaboration with the *British Journal of Haematology* and the *American Journal of Hematology* and it also attracts independent contributions and submissions directly to the journal.

Herein, this first issue covers a broad spectrum of important epidemiologic, diagnostic, therapeutic, and prognostic topics within haematology including lymphoid malignancies, myeloid neoplasms, plasma cell neoplasms, immunotherapy and cellular therapy, and also a host of benign haematologic subjects. Manuscripts in this first issue include prospective clinical trial results, quality of life analyses, meta‐analyses and retrospective “real‐world” data sets, as well as innovative biomarker and translational analyses. Furthermore, manuscript formats include full original articles, reviews, brief reports, case reports, and special images in haematology.

To highlight several manuscripts included in this issue of *eJHaem*, Gunther et al from MD Anderson Cancer Center reported on a large retrospective “real‐world” analysis of the impact of partial bleomycin omission in patients with early‐stage Hodgkin lymphoma; Pollyea et al delineated the spectrum of diagnostic and molecular testing patterns in patients with untreated acute myelogeous leukemia from the Connect^®^ MDS/AML Disease Registry; Premkumar et al performed a large scale meta‐analysis regarding the use of daratumumab in high‐risk multiple myeloma; Ishiyama et al reported on the impact of a PNH‐phenotype in response to eltrombopag in patients with refractory aplastic anemia; Girdlestone et al analyzing the milieu of immune reconstitution following umbilical cord blood transplantation in United Kingdom paediatric patients; and Sharman et al for the Connect CLL Registry reported on longitudinal health‐related quality of life for first‐line treated chronic lymphocytic leukemia patients.

In addition, the *eJHaem* is proud to have established an early track record for rapid and timely assessment of submitted papers. Among the first 100 manuscripts examined by the *eJHaem*, the median time of submission to first decision was 12.6 days and the median time for submission to acceptance was 17.6 days. We are thankful to the associate editors of *eJHaem* for their outstanding collaboration and nimble assessments as well as to the dedication of all peer reviewers who have helped to ensure high‐quality content and efficient throughput in an author‐centric fashion.

Altogether, *eJHaem* is honoured to provide the international haematology community with this first issue. We look forward to continuing to work closely with authors in order to publish haematology content that is timely, innovative, scientifically strong, and high impact.

Andrew M. Evens, DO, MSc, FACP

Editor‐in‐Chief, *eJHaem*

Professor of Medicine, Rutgers Robert Wood Johnson Medical School

Associate Director for Clinical Services, Rutgers Cancer Institute of New Jersey

Director, Lymphoma Program, Division of Blood Disorders

Medical Director, Oncology Service Line, RWJBarnabas Health

